# First-in-Human Study of MDG1011, a TCR-T Therapy Directed Against HLA-A*02:01-Restricted PRAME Antigen for High-Risk Myeloid and Lymphoid Neoplasms

**DOI:** 10.3390/cancers17182968

**Published:** 2025-09-11

**Authors:** Simone Thomas, Martin Wermke, Vladan Vučinić, Eva Wagner-Drouet, Andreas Mackensen, Robert Zeiser, Gesine Bug, Michael Schmitt, Wolfgang Herr, Petra U. Prinz, Maja Bürdek, Silke Raffegerst, Anna Tafuri, Christiane Geiger, Kirsty Crame, René Goedkoop, Kai Pinkernell, Dolores J. Schendel

**Affiliations:** 1 Department for Internal Medicine III, Hematology and Internal Oncology, University Hospital Regensburg, 93053 Regensburg, Germany; 2 Leibniz Institute for Immunotherapy, 93053 Regensburg, Germany; 3 Bavarian Center for Cancer Research (BZKF), 91054 Erlangen, Germany; 4 National Center for Tumor Diseases, University Hospital Carl Gustav Carus TU Dresden, 01307 Dresden, Germany; 5 Department of Hematology, Cell Therapy and Hemostaseology, University Leipzig Medical Center, 04103 Leipzig, Germany; 6 Department of Haematology and Medical Oncology, University Medical Center Mainz, 55131 Mainz, Germany; 7 Department of Internal Medicine 5, Hematology and Oncology, University Hospital Erlangen, 91054 Erlangen, Germany; 8 Department of Medicine I, Hematology, Oncology and Stem-Cell Transplantation, University of Freiburg Medical Center, 79110 Freiburg, Germany; 9 Department of Medicine 2, University Hospital, Goethe University Frankfurt, 60596 Frankfurt, Germany; 10 Internal Medicine V, Hematology, Oncology and Rheumatology, University Hospital Heidelberg, 69120 Heidelberg, Germany; 11 Medigene AG, 82152 Planegg-Martinsried, Germany

**Keywords:** T cell receptor, TCR-T therapy, PRAME, phase I clinical trial, hematological malignancies, AML, MDS, MM

## Abstract

Background/objectives: MDG1011 is a Preferentially Expressed Antigen in Melanoma (PRAME)-specific autologous T cell receptor (TCR) T cell therapy for HLA-A*02:01-positive patients. Data from the first-in-human (FIH) clinical trial, CD-TCR-001, are reported here regarding treatment feasibility, safety, tolerability, and clinical activity of MDG1011 in patients with relapsed/refractory (r/r) acute myeloid leukemia (AML), myelodysplastic syndrome (MDS), and multiple myeloma (MM). Methods: Nine of thirteen enrolled patients received MDG1011 at dose levels ranging from 0.1 to 5 × 10^6^ TCR-T cells per kg body weight. In addition to clinical assessments, immune monitoring of cytokines associated with cytokine release syndrome (CRS), presence and persistence of MDG1011, and changes in levels of PRAME mRNA were used to assess safety and potential biological activity at defined time points. Results: The treatment was well tolerated. No dose-limiting toxicities (DLTs) were observed, and the most common serious adverse events were associated with lymphodepleting chemotherapy and/or disease progression. Various parameters, such as measurable clinical responses in two patients, the occurrence of CRS in two additional patients, and reductions in PRAME mRNA levels in bone marrow (BM) or peripheral blood (PB) in seven patients, served as signs of the clinical and biological activity of MDG1011 TCR-T therapy. Conclusions: Patients enrolled in the phase 1 part of CD-TCR-001 displayed signs of potential clinical and biological activity of MDG1011 among the small number of patients studied. Advanced disease stage and rapid progression in the r/r AML patients limited clinical impact. The acceptable safety profile of MDG1011 merits further investigation of this TCR-T therapy, potentially in patients at an earlier stage of their disease and with lower tumor burden.

## Simple Summary

Adoptive cell therapy (ACT) using chimeric antigen receptor-engineered T cells (CAR-T) has been highly successful in the long-term clinical control of diverse B cell malignancies and multiple myeloma (MM). Expression of suitable target antigens by cancer cells that direct specific CAR-T recognition underlies this success. ACT for other cancers requires the identification of specific and safe target antigens. T cell receptor-engineered T cells (TCR-T) offer a potential solution based on recognition of peptide epitopes derived from overexpressed or mutated intracellular proteins presented by HLA molecules at the tumor cell surface. Preferentially Expressed Antigen in Melanoma (PRAME) may be well suited for TCR-T therapy of acute myeloid leukemia (AML) and myelodysplastic syndrome (MDS) through its broad overexpression in leukemic blasts of myeloid origin. MDG1011 TCR-T cell therapy, with specificity for an HLA-A2-presented, PRAME-derived peptide, was developed for first-in-human (FIH) phase 1 safety assessment in HLA-A2^+^ patients with PRAME^+^ AML, MDS, and multiple myeloma (MM).

## 1. Introduction

MDG1011 is a gene-modified adoptive cell therapy (ACT) using patient-derived autologous CD8^+^ T cells engineered to express a T cell receptor (TCR) recognizing the cancer-testis antigen Preferentially Expressed Antigen in Melanoma (PRAME). The specific, sensitive, and safe (3S) TCR used in MDG1011 recognizes the PRAME_100–108_ epitope (VLDGLDVLL) presented by the HLA-A*02:01 allotype, designated as the VLD-TCR. It was isolated using an in vitro priming approach as described [1,2]. Extensive nonclinical analysis supported the rationale for the first-in-human (FIH) use of MDG1011 TCR-T therapy [3].

PRAME was chosen as the target antigen based on several features: it is frequently expressed in leukemic blasts of lymphoid and myeloid origin, but displays a strong safety profile, with higher PRAME expression in healthy tissues limited to testis tissue and mature dendritic cells (DCs) [2]. PRAME is present in spermatocytes (unpublished observations), which are protected from TCR recognition by missing HLA class I expression [4]. PRAME plays a role in hematopoietic differentiation and apoptosis and may be essential for cell survival, potentially allowing counter-selection against PRAME antigen-loss by immune selection [5]. Moreover, targeting PRAME with TCR-T cells in solid tumors has recently been reported to be safe and well tolerated in a phase I study [6].

PRAME expression has been documented in numerous studies of AML, with frequencies ranging from 30 to 65% of mRNA samples, as recently reviewed [5]. One of the largest studies of 2000 pediatric and adult patients showed a high prevalence of PRAME in AML [7]. Increased levels of PRAME have been associated with improved prognosis [5]. In one study, a DNA microanalysis of samples from adult AML patients showed a trend for high levels of PRAME mRNA to correlate with improved overall survival (OS) [8]. In addition, PRAME-specific T cells in AML patients who received hematopoietic stem cell transplantation (HSCT) were associated with long-term survival [9,10,11], indicating the suitability of PRAME for T cell recognition and leukemia control. PRAME expression in MDS has not been studied as systematically as in AML, but it has been reported in around 75% of patients diagnosed with MDS [12]. Results from one study suggest PRAME expression as a negative prognostic factor in MDS patients with low BM blasts (less than 5%), classified into the lower IPSS-R risk category [13]. In MM patients, the expression of PRAME in BM cells and sorted plasma cells showed greater variations [14,15,16]. PRAME overexpression in the BM was reported as an adverse prognostic factor of progression-free survival in patients not treated with bortezomib [15,16], but bortezomib treatment can improve outcomes in these patients [17]. In total, the prevalence of PRAME expression forms the rationale for the development of TCR-T immunotherapy targeting PRAME in these three hematological malignancies.

MDG1011 was developed for use in a multicentre, non-randomized, open-label phase I/II clinical trial, CD-TCR-001 (NCT03503968; EudraCT 2017-000440-18), in patients with relapsed/refractory AML (r/rAML), MDS, or MM. The results of the phase I part of the CD-TCR-001 trial are reported here, with concordant assessments of biological activity in the patients treated with MDG1011.

## 2. Materials and Methods

### 2.1. IMP Manufacture

Investigational medicinal products (IMPs) consisted of subject-derived autologous CD8^+^ T cells, enriched by CD8-immunomagnetic bead isolation from unstimulated leukapheresis of mononuclear cells, followed by cryopreservation. CD8^+^ cells were thawed, stimulated with anti-CD3/anti-CD28 antibodies, and transduced with the self-inactivating (SIN) γ-retroviral vector encoding the VLD-TCR. Expanded cells were harvested and cryo-preserved. Retroviral vector production, IMP manufacturing, and QP release were performed at a contract development and manufacturing organization (CDMO) (BioNTech IMFS GmbH, Idar-Oberstein, Germany) and required 5–7 weeks. Extensive characterization of all MDG1011 IMPs has been detailed elsewhere [3].

### 2.2. Clinical Study Design

The FIH study of MDG1011 was designed as a multicentre, non-randomized, open-label, phase I/II clinical trial, carried out at nine study sites in Germany. The primary objectives of the phase I part of CD-TCR-001 were to assess the safety and tolerability of MDG1011, establish the maximum tolerated dose (MTD) and/or recommended phase II dose (RP2D), and assess the percentage of patients who received the planned doses of MDG1011.

The population eligible to receive MDG1011 consisted of patients with high-risk r/rAML, MDS, or MM. The inclusion/exclusion criteria can be found online (https://www.clinicaltrialsregister.eu/ctr-search/trial/2017-000440-18/DE (accessed on 30 July 2025)). Patient selection was based on the presence of the HLA-A*02:01 genotype, determined by next-generation or Sanger sequencing, and the detection of PRAME mRNA in BM or PB samples using quantitative real-time polymerase chain reaction (qPCR) performed at IMGM Laboratories GmbH (Munich, Germany). Patients classified as double-positive for HLA-A*02:01 and PRAME were assessed clinically for trial eligibility. Approved leukapheresis procedures were used to collect samples from enrolled patients at the study sites and shipped to the central CDMO for IMP production. The vein-to-vein time for MDG1011 therapy was on average 8.3 weeks (range 7.0–11.8 weeks). All patients received lymphodepleting chemotherapy (LDC) (cyclophosphamide 300 mg/m^2^ and fludarabine 25 mg/m^2^ intravenously on days −5 to −3) prior to a single intravenous infusion of IMP. Bridging therapy was allowed between leukapheresis and LDC at the investigators’ discretion.

**Figure 1:**
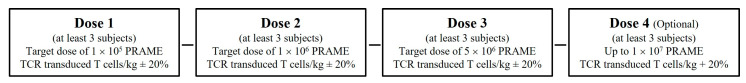
Dose cohort design. Classical 3+3 dose escalation study design to determine maximum tolerated dose (MTD) and recommended phase 2 dose (RP2D). Three dose levels were investigated: 0.1 × 10^6^, 1 × 10^6^, and 5 × 10^6^ PRAME (VLD)-TCR-expressing T cells per kg body weight. More than one patient with acute myeloid leukemia (AML)/myelodysplastic syndrome (MDS) was required per dose cohort.

Patients were enrolled into one of three dose cohorts intended to establish the maximum tolerated dose (MTD) or recommended phase 2 dose (RP2D), with an option to include a fourth dose cohort. Each dose was based on VLD-TCR-expressing T cells per kg body weight of the patient at screening and ranged from 0.1 × 10^6^ for cohort 1, 1 × 10^6^ for cohort 2 to 5 × 10^6^ for cohort 3 (Figure 1).

Dose escalation followed a 3 + 3 cohort design, including at least 3 initial patients per dose level and then 3 additional patients to be enrolled in case of a DLT event, as listed in Supplementary Table S1. The DLT evaluation period covered 28 days, starting from the day of IMP administration (day 0).

The CD-TCR-001 primary endpoints were as follows: (1) incidence and severity of adverse events [AEs] at 3 months, according to the Common Terminology Criteria for Adverse Events (NCI CTCAE v4.0), (2) MTD and/or RP2D of IMP measured by DLTs up to 28 days post-infusion, and (3) feasibility, defined as the percentage (%) of patients that received the planned target dose of IMP.

The trial was approved by the responsible Ethics Committee of the University of Regensburg (number 17-683-112), the independent ethics committee of each study site, and the first-in-country trial approval by the Paul-Ehrlich Institute, Federal Institute for Vaccines and Biomedicines, Langen, Germany. All patients provided their written informed consent. The trial was conducted in compliance with the principles of the Declaration of Helsinki.

### 2.3. Statistical Analysis

Analyses were performed on the Safety Population (SAF), consisting of all patients who underwent leukapheresis. Analysis of AEs was also performed on patients who received MDG1011, for whom at least one valid post-infusion evaluation was available (SAF-MDG1011). Based on a small sample size, only descriptive analysis was performed. The following parameters were used to summarize the data: quantitative variables included mean, 95% confidence interval (CI) of the mean, standard deviation (SD), minimum, quartiles, maximum, and number of available and number of missing observations. Ordinal and nominal variables included the number and percentage for each of the scores or categories, and the number of observations. Data analysis employed SAS software v9.4 (SAS Institute Inc., Cary, NC, USA).

### 2.4. Immune Monitoring

Biological assessments of MDG1011 were made with PB and/or BM samples obtained at defined time points before and after IMP infusion. Samples of each patient were assessed in two groups for better comparison: group 1 included samples collected up to week 4 after IMP infusion, and group 2 encompassed samples from week 4 to the end-of-trial (EoT) visit. An exception was made for patient C3P3, whose samples were analyzed individually after week 4.

PRAME mRNA expression was determined by qPCR with cDNA samples generated from either 250 ng or 500 ng RNA extracted from PAXgene Blood and/or Bone Marrow RNA tubes (Qiagen, Hilden, Germany) as described [3].

MDG1011 present in post-infusion samples was assessed by flow cytometry (FC) of PB-derived mononuclear cells (PBMCs) using antibodies detecting CD45 (clone HI30), CD3 (clone UCHT1), CD4 (clone SK3), CD8 (clone RPA-T8) (all BD Biosciences, Franklin Lakes, NJ, USA), TRBV9 (clone BL37.2, Beckman Coulter, Brea, CA, USA), and HLA-A*02:01/PRAME-VLD dextramer (Immudex, Copenhagen, Denmark). Cell acquisition was performed using the LSR Fortessa flow cytometer, and data were analyzed with FlowJo software version 10 (both BD Biosciences).

Molecular detection of MDG1011 was made using droplet digital PCR (dPCR) for the RNA quantification of the viral element Woodchuck Hepatitis Post-Transcriptional Regulatory Element (WPRE) included in the VLD-TCR gene construct, with GUSB as a reference gene. RNA transcripts isolated from lysed PBMCs were converted into cDNA (First Strand cDNA Synthesis Kit, Roche, Basel, Switzerland) and used for PCR amplification with the Naica system (Stilla Technologies, Villejuif, France).

Cytokines associated with CRS were analyzed in sequential serum samples and included interferon (IFN)-γ, interleukin (IL)-6, soluble glycoprotein 130 (sgp130), monocyte chemoattractant protein-1 (MCP-1), and soluble IL-6 receptor (sIL-6R). Sample analysis was conducted at Eurofins Central Laboratory (Breda, The Netherlands) using the Magpix system (Luminex, Austin, TX, USA) and Millipore assay kits (HCYTOMAG-60K, HSCRMAG-32K, Merck KGaA, Darmstadt, Germany), validated according to manufacturers’ instructions.

## 3. Results

### 3.1. Patient Disposition

**Figure 2:**
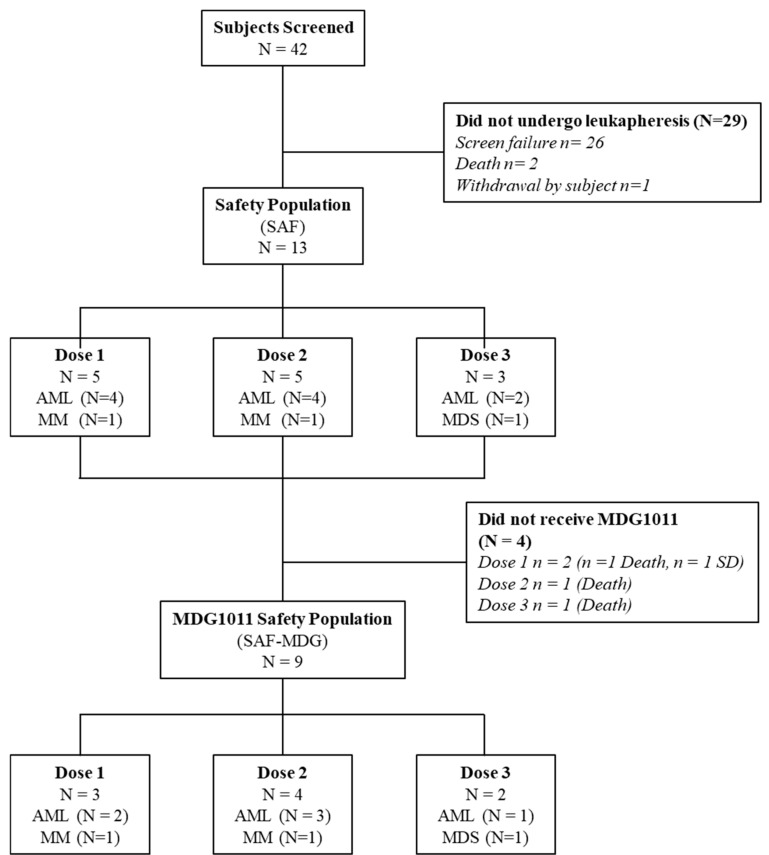
Disposition of patients. Nine patients were treated with MDG1011 at the three escalating dose levels (N = 3, 4, and 2 patients, respectively); 3 patients succumbed to their disease before investigational medicinal product (IMP) infusion, and 1 patient did not receive IMP infusion due to stable disease (SAF: safety population).

Between April 2018 and July 2021, 42 patients were screened for HLA genotype and PRAME expression as part of the screening study MDG-TA-001 or within the CD-TCR-001 trial. This was followed by clinical assessment for study eligibility (Figure 2). Of these 42 patients, 29 did not undergo leukapheresis due to 26 screening failures, 2 deaths, and 1 withdrawal of consent to participate in the study. Thirteen patients double-positive for HLA-A*02:01 and PRAME underwent leukapheresis and constituted the Safety Population (SAF). Four of thirteen patients did not receive MDG1011 due to progressive disease (*n* = 3) or stable disease (*n* = 1). Thereby, the SAF-MDG1011 consisted of 9 patients who received MDG1011, including 3 in cohort 1, 4 in cohort 2, and 2 in cohort 3.

**Figure 3:**
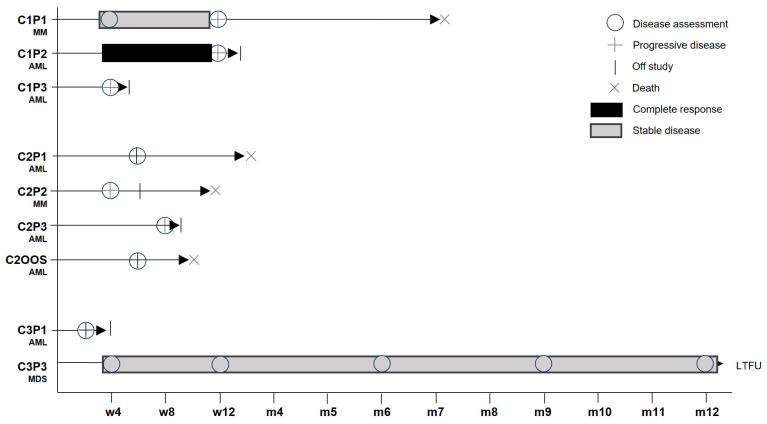
Time points at which patients experienced disease assessment, disease progression, or death. Time periods of complete response or stable disease for respective patients are shown using dark and light gray bars, respectively.

One patient with MDS and myelofibrosis completed the 12-month study period without progression to secondary AML (Figure 3).

Recruitment of eligible patients was strongly hampered during the peak periods of the COVID-19 pandemic, to the extent that recruitment was put on hold in February 2022 and subsequently halted in July 2022. Consequently, the phase 2 part of CD-TCR-001 was not initiated.

### 3.2. Baseline Characteristics and Demographics

The median age of patients undergoing leukapheresis (SAF) was 65.0 years (range 55–80 years). Six patients were male (46.2%), and seven were female. All patients were Caucasian. Patients with AML were the most frequent (*n* = 10). Five of the nine patients infused with MDG1011 were heavily pre-treated with ≥3 lines of therapy, including allogeneic or autologous HSCT. Six patients required bridging therapy between leukapheresis and MDG1011 infusion. Baseline patient characteristics are listed in Table 1.

For 10 patients with AML, the mean time after first diagnosis was 20.5 months (SD 13.0 months; range 5–44 months). The WHO classification for three patients was AML with MDS-related changes. Half of the patients presented with de novo AML and half with secondary AML. Most patients (62.5%) had no history of extramedullary disease at diagnosis, and most (75.0%) were in an adverse risk category at presentation. One patient with a WHO classification of MDS with multilineage dysplasia had a time of 62 months since histological diagnosis. At the time of study inclusion, he was no longer responding to ongoing therapy, and peripheral blood blast counts were increasing. Mean time from histological diagnosis for the two patients with MM was 20 and 48 months, respectively; both presented with Durie–Salmon stage III disease, with subclassification A. Adequate major organ function was part of the CD-TCR-001 in-/exclusion criteria. No clinically significant cardiac, pulmonary, renal, or liver function outside of normal range was observed for patients who received the IMP.

### 3.3. IMP Manufacturing Feasibility, Treatment Feasibility, and IMP Exposure

IMP manufacture was successful for 12 of 13 patients (92.3%). Leukapheresis was performed twice for two patients in cohort 3; one leukapheresis sufficed for all other patients. On average, total white blood cell count (WBC) from leukapheresis was 62.5 × 10^9^ cells (SD 82.1; range 2–213 × 10^9^ cells). IMPs comprised 14–49% VLD-TCR-expressing CD8^+^ T cells; T cell subset composition was variable, but all IMPs contained Tscm and Tcm cells, reaching up to 72% of CD8^+^ T cells [3]. All IMPs showed IFN-γ secretion and target cell killing after stimulation with antigen-presenting cells pulsed with exogenous VLD peptides, or in response to tumor cell lines expressing endogenous PRAME and HLA-A2 [3]. In total, 9 of 13 patients (69.2%; SAF-MDG1011) were treated with MDG1011: 3 received dose 1, 4 received dose 2, including 1 out-of-specification (OOS) product, and 2 received dose 3. Optional dose 4 was not investigated, notwithstanding one patient who entered screening for cohort 4 who did not move forward since the decision was made to halt the study. Three patients died before IMP was available for administration, and one patient with stable disease was not treated. The four patients who did not receive the IMP did not receive LDC.

### 3.4. Biological Activity

Three immune monitoring assessments were made before and after IMP infusion: (1) changes in PRAME mRNA levels in BM or PB samples, (2) presence and persistence of MDG1011 in sequential PB samples, and (3) alterations in CRS-associated proteins in serial serum samples. Leukapheresis starting materials were assessed for cellular content and compared to the composition of the infused IMPs, as shown elsewhere [3]. The frequency of CD34^+^ cells was determined in starting materials and varied from background values for the two MM patients to frequencies ranging from 0.86 to 82.2% for the AML and MDS patients (Table 2). CD34^+^ cells were rare (<0.14%) in healthy donor leukapheresis controls used for chemistry, manufacturing, and controls (CMC) development (unpublished observations), indicating an association of CD34^+^ cells with disease in patient starting materials. Despite substantial frequencies of CD34^+^ cells in starting materials of several patients, CD34^+^ cells were not seen in any final IMP [3]. This was deemed critical to diminish the risk of secondary neoplasms due to the mutagenic potential of gamma retroviruses for CD34^+^ stem cells [18,19]. Only one IMP failed to meet all release specifications, based on elevated CD4^+^ and reduced CD8^+^ cell percentages, according to the specified criteria. Nevertheless, the OOS IMP was applied to the AML patient in cohort 2, designated as patient C2OOS, following documented agreement of the investigator and subsequent QP release of the IMP under an OOS procedure.

PRAME mRNA expression in BM or PB was compared before and after IMP infusion to assess the impact of MDG1011 on tumor burden (Table 2).

**Figure 4:**
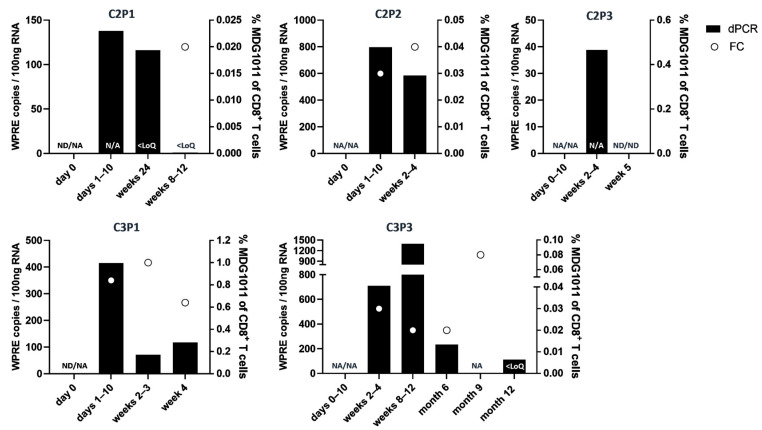
In vivo persistence of MDG1011 in peripheral blood. Peripheral blood mononuclear cells (PBMCs) were isolated from patient blood samples to detect MDG1011. For the assessment of the viral element Woodchuck Hepatitis Post-Transcriptional Regulatory Element (WPRE), RNA was extracted, converted into cDNA, and analyzed using droplet digital PCR (dPCR). The frequency of MDG1011 was evaluated by flow cytometry (FC), employing antibodies against TRVB9 and HLA-A*02:01/PRAME-VLD dextramer for detection. Represented is data of 5 patients from cohorts 2 and 3, whose samples showed presence and persistence of MDG1011 cells in sequential visits after IMP infusion. The left *y*-axis displays the number of WPRE copies per 100 ng of RNA, represented as black bars; the right *y*-axis shows the percentage of MDG1011 T cells among the CD8^+^ T cell population, indicated by white circles, at various time points indicated on the *x*-axis. ND = not detectable; NA = no analysis due to insufficient material; <LoQ = Limit of Quantification (dPCR:36; FC 0.015%).

Substantial changes were detected in most patients comparing samples from screening (SCR) and week 4 visits. Increases were seen in AML patient C1P2 and MM patient C2P2, while decreases from 8 to 96% were seen in the others. Flow cytometry (FC) and molecular dPCR were used to detect circulating MDG1011 cells in sequential PB samples after IMP infusion. Sporadic positive signals were detected in the limited samples of sufficient quality available for several patients in cohort 1 and cohort 2 (Supplementary Table S2). Contamination of PB by circulating leukemic blasts negatively impacted sample quality in AML patients who experienced rapid disease progression, limiting assessments due to low numbers of T cells. MDG1011 was not consistently detected in PB based on the low dose (0.1 × 10^6^ VLD-TCR-T^+^ cells/kg body weight) applied in cohort 1. Stronger cellular and molecular signals were seen in patients in cohort 2 and cohort 3, demonstrating that the dual assay approach was suited to track VLD-TCR-expressing T cells in patients receiving higher amounts of MDG1011 (Figure 4).

Patient C3P3 with multilineage MDS and myelofibrosis remained on study through month 12, with MDG1011 persistence seen over one year.

Protein markers associated with acute inflammation seen with CRS were assessed in sequential serum samples and varied substantially (Supplementary Table S3) [20]. IL-6, a key player in CRS [21], was undetectable or low in patients of cohort 1. It was elevated shortly in patient C2P1 at visit V2; however, consistent increases were not observed in subsequent samples. This patient was reported to have symptoms of grade 1 CRS. Low levels were seen on occasion in the other two AML patients of cohort 2, but IL-6 was below detection in the MM patient of cohort 2. The highest levels of IL-6, persisting over time, were seen in patient C3P1, who manifested symptoms of grade 2 CRS.

### 3.5. Clinical Activity

Clinical activity of different duration was reported for two patients (Figure 3). Patient C1P2 with relapsed AML and extramedullary disease was reported to have a complete response (CR) defined by the absence of circulating blasts at day 28 (where BM assessment was not available) after receiving IMP at dose level 1. While MDG1011 cells were below levels of quantification in the PB of this patient, a population of PRAME_VLD_ multimer-positive cells arising from the natural T cell repertoire was found in PB (unpublished observations). Disease progression was reported at day 84 after IMP infusion.

Patient C3P3 with multilineage MDS and myelofibrosis treated at dose level 3 completed the 12-month study period without progression to secondary AML. PRAME mRNA levels decreased by 96% in samples of this patient between the screening and week 4 visits. This patient had two serious treatment-emergent adverse events (TEAEs), comprising persistent decreased white blood cell counts and febrile neutropenia, both requiring prolonged hospitalization. MDG1011 persistence in patient C3P3 was seen by FC and dPCR over the 12-month study period (Figure 4, Supplementary Table S2).

The best response reported for patient C1P1 with MM was a temporary stable disease until progression of disease on day 84 after IMP infusion. All other patients did not respond to MDG1011 treatment and showed early progressive disease (Figure 3).

### 3.6. Safety

Among the nine patients treated with MDG1011, no DLTs were observed. CRS of grade 1 or grade 2 occurred in AML patients C2P1 (starting on day 3 after IMP infusion) and C3P1 (starting on day 2 after IMP infusion), respectively. MDG1011 was detected in both patients at multiple time points after IMP infusion (Figure 4). The grade 1 CRS resolved with symptomatic treatment alone, whereas the patient with grade 2 CRS was treated with intravenous anti-IL-6R monoclonal antibody (tocilizumab), fresh frozen plasma packs, and fluid therapy. This patient had the highest levels of PRAME at SCR and Week 4 among all patients analyzed (Table 2). MDG1011 in patient C3P1 reached a level of 1% CD8^+^ T cells two weeks after IMP infusion. Immune effector cell-associated neurotoxicity was not reported. TEAEs, defined as events which started on or after the date of leukapheresis, are listed in Table 3.

For the entire study, 124 TEAEs were reported in 13 patients (100.0%), 31 in 6 patients (46.2%) related to LDC, and 23 in 9 patients (69.2%) due to disease progression. In eight patients (61.5%), TEAEs led to death, none of which were related to MDG1011, including the four patients who did not receive MDG1011. In the SAF-MDG1011 population, 68 TEAEs were reported for the nine treated patients: 12 serious TEAEs in seven patients (77.8%) and 26 TEAEs of toxicity grade 3 or higher in eight patients (88.9%). Six TEAEs were considered of special interest: CRS, neutropenic sepsis, neuralgia (occurred before MDG1011 administration), multi-organ dysfunction syndrome, septic shock, and pyrexia. TEAEs leading to death occurred for four patients (44.4%): one in the MDG1011 dose cohort 1, and three in dose cohort 2. These were all considered not related to the LDC regimen and not related to MDG1011. Eighteen TEAEs related to MDG1011 were reported for six patients (66.7%) (Table 4).

The most frequent MDG1011-related TEAEs were CRS and aspartate aminotransferase elevation, each observed in two patients. No patterns were observed between dose cohorts in the assessment of TEAEs by preferred term, system organ class, or bridging therapy. Life-threatening grade 4 toxicities were reported for two patients: infusion-related reaction after IMP administration in one patient in cohort 2 and leukopenia in one patient in cohort 3. For the other patients with related TEAEs, the toxicity grade was mild or moderate. No fatal TEAEs (Grade 5) were considered related to MDG1011. One TEAE reported led to the discontinuation of fludarabine. No DLTs or TEAEs leading to the hold or discontinuation of CD-TCR-001 were reported. A detailed listing of TEAEs is given in Supplementary Table S4.

## 4. Discussion

MDG1011 was developed as a TCR-T therapy for patients with r/rAML, MDS, and MM, who were double-positive for HLA-A*02:01 and PRAME. At the time this first-in-country study was initiated in Germany, the development of safe and effective therapies was highly desirable for patients suffering from r/rAML, MDS, and MM, as no other T cell immunotherapies (e.g., CAR-T cells) were available.

Thirteen patients were included in the phase 1 part of the CD-TCR-001 trial, and IMP was successfully manufactured for 12 of 13 patients with a success rate of 92.3%, demonstrating an unexpectedly high feasibility given the disease status, extensive history of chemotherapy, and age of many patients in the study. Nine of thirteen patients received MDG1011, demonstrating a treatment feasibility of 69.2%. One patient with stable disease was not treated. Three patients succumbed to disease before LDC and IMP infusion, reflecting fulminant disease and the short treatment window in this advanced patient population.

At the time of study initiation, limited experience from CAR-T therapies in hematologic malignancies and a few TCR-T trials showed that treatment was associated with a relatively high risk of potentially life-threatening CRS and other inflammatory side effects [22,23] or toxicity due to prior unknown cross-reactivities of TCR-T cells for healthy cells [24,25]. In light thereof, an ultra-low starting dose of 0.1 × 10^6^ VLD-TCR-expressing T cells per kg body weight was applied using a starting cell number estimated to be one log below an expected therapeutic dose. This ultra-low starting dose posed a significant hurdle for the recruitment of end-stage cancer patients, given the known toxicities of LDC and low expectation for clinical benefit at the first dose level.

Clinical activity was seen for two patients. Patient C1P2 with r/rAML was reported to have an absence of circulating blasts at day 28, which was transient as disease progression was seen at day 84. MDG1011 T cells were not detected and not expected, given the low number of cells infused in this patient. As patient C1P2 entered the trial post-HSCT relapse, the response may have been influenced by the effects of LDC or the transient perturbation of the immune system by MDG1011. Patient C3P3, the only MDS patient treated with MDG1011, showed reductions in PRAME mRNA levels by 96%, as quantified in PB between screening and day 28. Assessment of BM samples was not possible in this patient due to inadequate sample acquisition based on extensive myelofibrosis. This patient did not progress to secondary AML during the 12 months of study. Sequential PB samples showed long-term persistence of MDG1011 cells in patient C3P3, including the EoT visit at one year.

CRS of grade 1 or grade 2 occurred in 2 AML patients treated in the higher dose cohorts (C2P1 and C3P1). As CRS is pathognomic for T cell therapies, this served as further evidence of the biological activity of MDG1011 [26,27]. MDG1011 was detected in both patients after IMP infusion, reaching a level of 1% of CD8^+^ TCR-positive T cells at two weeks in the patient with grade 2 CRS. Signs of biological activity were also seen in seven of nine patients by reductions in PRAME mRNA ranging from 8 to 96% in BM or PB samples quantified between screening and day 28 after IMP infusion. MDG1011 cells were detected at very low levels in PB samples in one cohort 1 patient, but at higher levels in three patients in cohort 2, and both patients in cohort 3.

There were no DLTs for MDG1011. The serious TEAEs reported were associated with pre-conditioning LDC using cyclophosphamide and fludarabine, effects well known from CAR-T and other TCR-T cell therapies. CRS seen in two patients was clearly related to MDG1011 and is a known toxicity associated with engineered ACT. In fact, CRS is judged as a biological sign of CAR-T therapy that is associated with CAR-T cell expansion [26,27]. Grade 1 CRS resolved rapidly for C2P1 after symptomatic treatment; C3P1 with grade 2 CRS required treatment with tocilizumab. This patient had the highest level of PRAME mRNA among all patients at day 28, suggesting that MDG1011 cells may have received particularly strong tumor cell stimulation after IMP infusion. The highest frequency of MDG1011 cells was also seen in patient C3P1; follow-up data on T cell persistence were not available as this patient withdrew from the trial.

Characterization of the IMPs of individual patients was previously published, detailing the phenotypes of the T cell subpopulations contained within the IMPs, including the stem cell memory (Tscm) and central memory (Tcm) T cell fractions compared with the composition of apheresis starting materials [3]. High variations were seen in the IMPs, reflecting to some degree their levels in the apheresis starting materials [3]. The low numbers of MDG1011 found in the first four weeks after treatment may be related to the fact that supplementary IL-2, which can drive early TCR-T proliferation, was not used in this study. Also, the fraction of Tscm and Tcm cells was variable, with the highest percentage seen in the IMP for the patient with MDS [3]. Interestingly, MDG1011 persistence was detected over the 12-month period this patient was on study. CAR-T studies and animal models have demonstrated that the prevalence of T cells in drug products with stem-like properties, associated with Tscm and Tcm subpopulations, correlates with better clinical responses [28,29,30,31]. Recent advances in IMP manufacture that shorten production times substantially increase the number of stem-like cells in drug products, providing IMP with greater capacities for proliferation and persistence and clinical efficacy [32].

Long-term persistence of TCR-T cells, in the absence of supplemental IL-2, was seen in the patient with MDS, supporting the contention that greater clinical benefit may be achieved in patients with lower disease burdens. MDG1011 might serve the needs of AML patients with minimal residual disease (MRD), following CR by chemotherapy. An earlier study using DC vaccines as maintenance therapy in AML patients in CR with MRD after chemotherapy, which included PRAME and Wilm’s Tumor 1 (WT1) as target antigens, demonstrated an overall survival at five years of 75%, with 70% of patients ≥ 60 years of age being long-term survivors. These patients received DC vaccines monthly over a period of one year to maintain T cell responses to guard against disease progression, suggesting that persistent T cell responses could impact MRD and disease activity [33]. Likewise, treatment of patients with MRD after HSCT, when disease burden is low but relapse risk is high, could be a potential clinical setting for MDG1011 TCR-T therapy. Finally, MDG1011 applied to prevent progression to secondary AML is hypothesized by the outcome for the MDS patient who received this TCR-T immunotherapy. Given the safety profile, use of higher doses of improved IMPs and in combination with IL-2 or other modalities, such as checkpoint antibodies, could be considered to improve MDG1011-based TCR-T immunotherapy.

## 5. Conclusions

The CD-TCR-001 clinical trial met several goals: treatment feasibility of more than 60% was reached, no DLTs were found at the three dose levels tested, and the single IMP infusion was generally well tolerated, all suggesting safe application of MDG1011 in these patients. Signs pointing to potential clinical activity were noted in two of nine patients, although inclusion of heavily pre-treated, end-stage patients with r/rAML likely precluded major clinical impact, as noted for other forms of ACT tested in r/rAML patients [11,34]. Reductions in PRAME mRNA levels in four of six r/rAML patients in cohorts 2 and 3, and the occurrence of CRS in two AML patients, indicated biological activity of MDG1011 applied at the two higher dose levels in patients with advanced r/rAML. Long-term persistence of TCR-T cells, in the absence of supplemental IL-2, was seen in the patient with multilineage MDS who did not progress to secondary AML in the 12-month study period. This observation supports the contention that greater clinical benefit may be achieved in patients with lower disease burdens.

## 6. Patents

D.J.S is a co-inventor on WO2017/216324 related to this work and filed by Medigene Immunotherapies GmbH.

## Figures and Tables

**Table 1:** Demographic data and disease entity (SAF and SAF-MDG1011 populations).

		Dose 1	Dose 2	Dose 3	All Patients
SAF		N = 5	N = 5	N = 3	N = 13
Age (years)					
	Median (min, max)	64.0 (58, 71)	60.0 (55, 77)	69.0 (65, 80)	65.0 (55, 80)
Age group n (%)					
	<65 years	3 (60.0%)	3 (60.0%)	-	6 (46.2%)
	≥65 years	2 (40.0%)	2 (40.0%)	3 (100%)	7 (53.8%)
Sex n (%)					
	Male	3 (60.0%)	2 (40.0%)	1 (33.3%)	6 (46.2%)
	Female	2 (40.0%)	3 (60.0%)	2 (66.7%)	7 (53.8%)
Disease entity n (%)		n’ = 5	n’ = 5	n’ = 3	n’ = 13
	Acute myeloid leukemia (AML)	4 (80.0%)	4 (80.0%)	2 (66.7%)	10 (76.9%)
	Myelodysplastic syndrome (MDS)	-	-	1 (33.3%)	1 (7.7%)
	Multiple myeloma (MM)	1 (20.0%)	1 (20.0%)	-	2 (15.4%)
**SAF-MDG1011**		**N = 3**	**N = 4**	**N = 2**	**N = 9**
Prior lines of treatment					
	1	-	1 (25%)	1 (50%)	2 (22.2%)
	2	-	1 (25%)	1 (50%)	2 (22.2%)
	≥3	3 (100%)	2 (50%)	-	5 (55.5%)
Prior HSCT					
		3 (100%	3 (75%)	1 (50%)	7 (77.7%)
Bridging therapy					
	Hydroxycarbamid	1 (33%)	-	1 (50%)	2 (22.2%)
	Cytarabine	-	1 (25%)	-	1 (11.1%)
	Mitoxantrone	-	1 (25%)	-	1 (11.1%)
	Gilteritinib	-	1 (25%)	-	1 (11.1%)
	Azacitidine	-	-	1 (50%)	1 (11.1%)
	Carfilzomib	-	1 (25%)	-	1 (11.1%)
	Radiotherapy	1 (33%)	-	-	1 (11.1%)

N = number of patients; n = number of patients in the category; n’ = number of patients with an assessment; Min = minimum; Max = maximum; HSCT = hematopoietic stem cell transplantation (autologous or allogeneic).

**Table 2:** PRAME mRNA expression in bone marrow or peripheral blood.

	Cohort 1			Cohort 2				Cohort 3	
Patient	C1P1	C1P2	C1P3	C2P1	OOS	C2P2	C2P3	C3P1	C3P3
Indication	MM	AML	AML	AML	AML	MM	AML	AML	MDS
IMP number	MAR-004	MAR-006	MAR-010	MAR-012	MAR-014	MAR-018	MAR-022	MAR-028	MAR-033
Blasts (%) (leukapheresis)	0%	13%	57%	1%	82%	0%	26%	7%	17%
Source	BM	PB	PB	BM	BM	BM	BM	PB	PB
PRAME mRNA at SCR	1134	4714	327	1891	975	<LoQ	1814	24,247	1797 ^b^
PRAME mRNA at Week 4	545	6607	300 ^a^	424	<LoQ	112	1059	11,087 ^a^	<LoQ ^b^
Change (%)	52% decrease	40% increase	8% decrease	78% decrease	93% decrease	60% increase	42% decrease	54% decrease	96% decrease

MM = multiple myeloma; AML = acute myeloid leukemia; IMP = investigational medicinal product; BM = bone marrow; PB = peripheral blood; SCR = screening. PRAME copies/25ng RNA detected by qPCR; Limit of Quantification (LoQ) = 70; ^a^ Data from EoT visit (week 4); ^b^ RNA from isolated PBMCs (PAXgene tubes not available for this patient).

**Table 3:** Summary of TEAEs (SAF and SAF-MDG1011).

	Onset After Beginning MDG1011 Administration (SAF-MDG1011)	Entire Study (SAF)
	N = 9	N = 13
Patients presenting with any	n (%) nae	n (%) nae
TEAE	9 (100.0%) 68	13 (100.0%) 124
TEAE related to MDG1011	6 (66.7%) 18	6 (46.2%) 21 ^a^
TEAE related to cyclophosphamide	5 (55.6%) 17	6 (46.2%) 30
TEAE related to fludarabine	4 (44.4%) 16	6 (46.2%) 30
TEAE related to LDC ^b^	5 (55.6%) 17	6 (46.2%) 31
TEAE related to clinical trial procedure other than MDG1011, cyclophosphamide, or fludarabine	3 (33.3%) 4	4 (30.8%) 7
Serious TEAE	7 (77.8%) 12	12 (92.3%) 28
TEAE of special interest	3 (33.3%) 6	4 (30.8%) 9
TEAE with toxicity grade ≥ 3	8 (88.9%) 26	13 (100.0%) 54
TEAE due to disease progression	6 (66.7%) 14	9 (69.2%) 23
TEAE leading to death	4 (44.4%) 5	8 (61.5%) 9

N = number of patients in the analysis set; n = number of patients in the category; nae = number of adverse events; TEAE = treatment-emergent adverse event.^a^ Three of these adverse events were recorded as starting before MDG1011 administration; ^b^ TEAE related to LDC is defined as TEAE related to cyclophosphamide and/or fludarabine.

**Table 4:** Patients with TEAEs related to MDG1011.

		Onset After Beginning MDG1011 Administration (SAF-MDG1011)			
		Cohort 1	Cohort 2	Cohort 3	All Patients
System Organ Class		N = 3	N = 4	N = 2	N = 9
Preferred Term		n (%) nae	n (%) nae	n (%) nae	n (%) nae
Any TEAE related to MDG1011		2 (66.7%) 2	2 (50.0%) 6	2 (100.0%) 10	6 (66.7%) 18
Grade 1—Mild		1 (33.3%)	1 (25.0%)	-	2 (22.2%)
Grade 2—Moderate		1 (33.3%)	-	1 (50%)	2 (22.2%)
Grade 3—Severe		-	-	-	-
Grade 4—Life-threatening		-	1 (25%)	1 (50%)	2 (22.2%)
Grade 5—Fatal		-	-	-	-
General disorders and administration site conditions		-	1 (25.0%) 1	1 (50.0%) 1	2 (22.2%) 2
	Fatigue	-	1 (25.0%) 1	-	1 (11.1%) 1
	Pyrexia	-	-	1 (50.0%) 1	1 (11.1%) 1
Immune system disorders		-	1 (25.0%) 1	1 (50.0%) 1	2 (22.2%) 2
	Cytokine release syndrome	-	1 (25.0%) 1	1 (50.0%) 1	2 (22.2%) 2
Investigations		1 (33.3%) 1	-	1 (50.0%) 7	2 (22.2%) 8
	Aspartate aminotransferase increased	1 (33.3%) 1	-	1 (50.0%) 1	2 (22.2%) 2
	Alanine aminotransferase increased	-	-	1 (50.0%) 1	1 (11.1%) 1
	Gamma-glutamyltransferase increased		-	1 (50.0%) 1	1 (11.1%) 1
	Platelet count decreased	-	-	1 (50.0%) 2	1 (11.1%) 2
	White blood cell count decreased	-	-	1 (50.0%) 2	1 (11.1%) 2
Blood and lymphatic system disorders		-	-	1 (50.0%) 1	1 (11.1%) 1
	Febrile neutropenia	-	-	1 (50.0%) 1	1 (11.1%) 1
Infections and infestations		1 (33.3%) 1	-	-	1 (11.1%) 1
	Oropharyngeal candidiasis	1 (33.3%) 1	-	-	1 (11.1%) 1
Injury, poisoning and procedural complications		-	1 (25.0%) 1	-	1 (11.1%) 1
	Infusion-related reaction	-	1 (25.0%) 1	-	1 (11.1%) 1
Nervous system disorders		-	1 (25.0%) 2	-	1 (11.1%) 2
	Dizziness	-	1 (25.0%) 1	-	1 (11.1%) 1
	Headache	-	1 (25.0%) 1	-	1 (11.1%) 1
Skin and subcutaneous tissue disorders		-	1 (25.0%) 1	-	1 (11.1%) 1
	Rash maculo-papular	-	1 (25.0%) 1	-	1 (11.1%) 1

N = number of patients in the analysis set; n = number of patients in the category; nae = number of adverse events; TEAE = treatment-emergent adverse event.

## Data Availability

Data are available from D.J.S. upon reasonable request and with certain limitations.

## Supplementary Materials

The following supporting information can be downloaded at: https://www.mdpi.com/article/10.3390/cancers17182968/s1, Table S1: Summary of Dose-Limiting Toxicities; Table S2: In vivo Persistence of MDG1011 in Peripheral Blood; Table S3: Analysis of Cytokines Associated with CRS in Patient Serum; Table S4: TEAEs by MedDRA SOC and PT reported in ≥15% of all patients.
