# Supplementary material for: First-in-Human Study of MDG1011, a TCR-T Therapy Directed Against HLA-A*02:01-Restricted PRAME Antigen for High-Risk Myeloid and Lymphoid Neoplasms
Source: Cancers (Basel). 2025 Sep 11;17(18):2968. doi: 10.3390/cancers17182968 (PMC12468345; doi:10.3390/cancers17182968)
Supplement: Supplementary file 1 [file cancers-17-02968-s001.zip › cancers-3822121-supplementary.pdf]

Supplementary Material

Supplementary Table 1 - Summary of Dose-Limiting Toxicities

DLT	Definition
**General**	The DLT-evaluation period covers 28 days from the day of IMP administration (visit 0 [week 0] until visit 5 [week 4]).
**Hematologic DLT**	Prolonged neutropenia or thrombopenia CTCAE grade ≥ 4 not resolved at day 28, newly developed and not attributable to underlying disease or lymphodepleting regimen
**Non-hematologic DLT**	Any CTCAE grade ≥ 3 toxicity were considered as DLT except from:<br>• CTCAE grade 3 Cytokine release syndrome (CRS) (CRS graded according to (Lee et al. 2019))<br>• CTCAE grade 4 CRS that does respond to treatment within 48 hours; (CRS graded according to (Lee et al. 2019))<br>• CTCAE grade 3 Aspartate aminotransferase (AST)/ Alanine aminotransferase (ALT) increase that does resolve to ≤ Grade 1 within 14 days<br>• CTCAE grade 3 nervous system disorders in the absence of CRS<br>• CTCAE grade 4 nervous system disorders in the absence of CRS that do respond to treatment within 48 hours<br>• CTCAE grade 3 TLS<br>• CTCAE grade 4 TLS that does resolve within 7 days<br>• Electrolyte abnormalities of CTCAE grade 3<br>• Electrolyte abnormalities of CTCAE grade 4 that do resolve to ≤ grade 2 within 72 hours with or without treatment<br>• Fatigue
**Other DLT**	Any other unacceptable toxicity in the view of the investigator and the DSMB.

CTCAE = Common Terminology Criteria for Adverse Events; DLT = dose-limiting toxicity.

Supplementary Table 2 - *In vivo* Persistence of MDG1011 in Peripheral Blood

Patient[a]	Visit	Days after V0	MDG1011 (%) by FC[b]	WPRE (copies) by dPCR[c]
**C1P1**	V0	0	ND	NA
	V1	2	NA	NA
	V2	7	0.02	NA
	V5	28	<LoQ	<LoQ
	V7	56	0.03	<LoQ
	V8	84	<LoQ	<LoQ
	V9	162	<LoQ	ND
**C1P2**	V0	0	0.03	NA
	V1	2	NA	NA
	V2	6	ND	NA
	V5	29	<LoQ	<LoQ
	V7	56	<LoQ	<LoQ
	V8	84	ND	ND
	EoT	93	0.02	ND
**C1P3**	V0	0	ND	NA
	V1	2	ND	NA
	V2	7	ND	ND
	EoT	28	<LoQ	ND
**C2P1**	V0	0	NA	ND
	V1	3	NA	137.8
	V2	7	WNCR	<LoQ
	V5	27	< LoQ	116.3
	EoT	42	WNCR	<LoQ
	V8[d]	80	0.02	<LoQ
**C2P2**	V0	0	NA	NA
	V1	2	NA	NA
	V2	7	0.03	795.0
	V5	28	0.04	585.0
**C2P3**	V0	0	NA	NA
	V1	2	NA	NA
	V2	7	NA	NA
	V3	14	NA	<LoQ
	V4	21	NA	38.8
	V5	28	NA	NA
	EoT	37	ND	ND
**C3P1**	V0	0	NA	ND
	V1	2	NA	415.0
	V2	7	0.27	184.3
	V2b	10	0.84	49.8
	V3	14	1.00	72.0
	V4	21	0.53	<LoQ
	UNS3	23	0.69	119.3
	EoT	29	0.64	117.5
**C3P3**	V5	27	0.03	710.0
	V7	56	0.02	1393.0
	V8	71	0.02	935.0
	V9	183	0.02	235.0
	V10	274	0.08	NA
	EoT	371	<LoQ	112.0

WNCR = within negative control range; ND = not detectable; NA = analysis not done as insufficient material available; EoT = end-of-trial; < LoQ = Limit of Quantification (dPCR:36; FC 0.015%); [a] Patient C2OOS was not analyzed as no sufficient material available; [b] Determined on CD8+ T cells; [c] Copies per 100ng RNA; [d] Follow-up visit.

Supplementary Table 3 - Analysis of Cytokines Associated with CRS in Patient Serum

Patient	Visit	Days after V0	IL-6 [pg/ml]	sIL-6R [pg/ml]	sgp130 [pg/ml]	MCP-1 [pg/ml]	IFN-γ [pg/ml]
C1P1	V0	0	<3	29674	303313	1278	<3
	V1	2	4	28473	324431	1120	<3
	V2	7	3	30018	308002	739	<3
	V5	28	<3	25908	267663	475	<3
	V8	84	<3	38946	293316	266	<3
	V9	162	<3	49980	328025	718	<3
C1P2	V0	0	33	15903	248251	1975	<3
	V1	2	<3	12350	204305	575	<3
	V2	6	<3	12647	236426	745	<3
	V5	29	<3	20195	306491	869	<3
	V8	84	<3	20475	87814	515	<3
	EoT	93	4	26870	123080	739	<3
C1P3	V0	0	<3	56812	225178	646	<3
	V2	7	<3	83038	240437	240	<3
	EoT	28	<3	85196	283822	133	<3
C2P1	V0	0	13	24972	141662	211	<3
	V1	3	11	21597	108322	127	<3
	V2	7	265	38803	99277	112	<3
	V5	27	<3	49625	131530	266	<3
	EoT	42	5	53251	221727	147	<3
	V8[a]	80	<3	61762	279888	161	<3
C2OOS	V0	0	3	22623	261924	1830	<3
	V1	2	7	20127	253340	1934	<3
	UNS	2	4	17653	270364	907	<3
	V5	28	<3	22518	280273	963	<3
	EoT	44	<3	19418	267225	1141	<3
C2P2	V0	0	<3	22007	194127	932	<3
	V1	2	<3	19212	159891	899	<3
	V2	7	<3	19201	180678	623	<3
	V5	28	<3	23405	190785	514	<3
C2P3	V0	0	23	18071	155715	1540	<3
	V2	7	16	23265	150692	1486	<3
	V2b	10	7	21781	123379	1210	<3
	V5	28	8	33162	110562	230	<3
	EoT	37	10	43254	118935	281	<3
C3P1	V0	0	9	21301	160834	740	<3
	UNS	2	9	17618	138409	854	<3
	UNS	3	NA	NA	NA	NA	NA
	V2	7	224	95232	156064	1047	<3
	V2	9	103	116697	143241	978	<3
	UNS	23	125	129019	203277	1272	<3
	EoT	29	116	119637	178100	1156	<3

Patient	Visit	Days after V0	IL-6 [pg/ml]	sIL-6R [pg/ml]	sgp130 [pg/ml]	MCP-1 [pg/ml]	IFN-γ [pg/ml]
C3P3	V0	0	6	14501	270405	280	<3
	V1	2	5	12369	248439	481	<3
	V2	7	8	12395	247060	548	<3
	V5	27	<3	15895	293658	1130	<3
	V8	71	<3	17836	293720	411	<3
	V9	183	<3	6330	34138	223	<3
	V10	274	<3	6429	35896	224	<3
	EoT	371	<3	6583	36616	215	<3

NA = analysis not done; EoT = end-of-trial; UNS = unscheduled visit; IL = interleukin; sIL = soluble interleukin; sgp130 = soluble glycoprotein 130; MCP-1 = Monocyte chemoattractant protein-1; IFN-γ = interferon-γ
[a] Follow-up visit

Supplementary Table 4 - TEAEs by MedDRA SOC and PT reported in ≥ 15% of all patients

	Onset Before MDG1011 Administration (SAF)				Onset After Beginning of MDG1011 Administration (SAF-MDG1011)				Entire Study (SAF)			
	**Cohort 1**	**Cohort 2**	**Cohort 3**	**All Patients**	**Cohort 1**	**Cohort 2**	**Cohort 3**	**All Patients**	**Cohort 1**	**Cohort 2**	**Cohort 3**	**All Patients**
**System Organ Class**	**N = 5**	**N = 5**	**N = 3**	**N = 13**	**N = 3**	**N = 4**	**N = 2**	**N = 9**	**N = 5**	**N = 5**	**N = 3**	**N = 13**
Preferred Term	n (%) nae	n (%) nae	n (%) nae	n (%) nae	n (%) nae	n (%) nae	n (%) nae	n (%) nae	n (%) nae	n (%) nae	n (%) nae	n (%) nae
Any TEAE	5 (100%) 16	5 (100%) 28	3 (100%) 12	13 (100%) 56	3 (100%) 18	4 (100%) 36	2 (100%) 14	9 (100%) 68	5 (100%) 34	5 (100%) 64	3 (100%) 26	13 (100%) 124
**Blood and lymphatic system disorders**	2 (40.0%) 2	1 (20.0%) 3	2 (66.7%) 2	5 (38.5%) 7	1 (33.3%) 8	1 (25.0%) 2	2 (100.0%) 2	4 (44.4%) 12	3 (60.0%) 10	2 (40.0%) 5	2 (66.7%) 4	7 (53.8%) 19
Anaemia	1 (20.0%) 1	1 (20.0%) 2	1 (33.3%) 1	3 (23.1%) 4	–	1 (25.0%) 2	–	1 (11.1%) 2	1 (20.0%) 1	2 (40.0%) 4	1 (33.3%) 1	4 (30.8%) 6
Thrombocytopenia	–	1 (20.0%) 1	1 (33.3%) 1	2 (15.4%) 2	1 (33.3%) 1	–	–	1 (11.1%) 1	1 (20.0%) 1	1 (20.0%) 1	1 (33.3%) 1	3 (23.1%) 3
**General disorders and administration site conditions**	1 (20.0%) 2	1 (20.0%) 1	1 (33.3%) 1	3 (23.1%) 4	–	3 (75.0%) 4	1 (50.0%) 1	4 (44.4%) 5	1 (20.0%) 2	4 (80.0%) 5	2 (66.7%) 2	7 (53.8%) 9
Fatigue	–	–	–	–	–	2 (50.0%) 2	–	2 (22.2%) 2	–	2 (40.0%) 2	–	2 (15.4%) 2
Pyrexia	–	–	1 (33.3%) 1	1 (7.7%) 1	–	–	1 (50.0%) 1	1 (11.1%) 1	–	–	2 (66.7%) 2	2 (15.4%) 2
**Infections and infestations**	2 (40.0%) 2	1 (20.0%) 3	–	3 (23.1%) 5	2 (66.7%) 2	3 (75.0%) 5	–	5 (55.6%) 7	3 (60.0%) 4	4 (80.0%) 8	–	7 (53.8%) 12
Bronchitis	–	–	–	–	1 (33.3%) 1	1 (25.0%) 1	–	2 (22.2%) 2	1 (20.0%) 1	1 (20.0%) 1	–	2 (15.4%) 2
Septic shock	–	1 (20.0%) 1	–	1 (7.7%) 1	–	1 (25.0%) 1	–	1 (11.1%) 1	–	2 (40.0%) 2	–	2 (15.4%) 2
**Investigations**	2 (40.0%) 3	3 (60.0%) 6	1 (33.3%) 2	6 (46.2%) 11	1 (33.3%) 3	1 (25.0%) 3	1 (50.0%) 7	3 (33.3%) 13	2 (40.0%) 6	3 (60.0%) 9	1 (33.3%) 9	6 (46.2%) 24
Alanine aminotransferase increased	1 (20.0%) 1	1 (20.0%) 1	–	2 (15.4%) 2	–	–	1 (50.0%) 1	1 (11.1%) 1	1 (20.0%) 1	1 (20.0%) 1	1 (33.3%) 1	3 (23.1%) 3
Aspartate aminotransferase increased	–	1 (20.0%) 1	–	1 (7.7%) 1	1 (33.3%) 2	–	1 (50.0%) 1	2 (22.2%) 3	1 (20.0%) 2	1 (20.0%) 1	1 (33.3%) 1	3 (23.1%) 4
Platelet count decreased	1 (20.0%) 1	–	1 (33.3%) 1	2 (15.4%) 2	–	1 (25.0%) 1	1 (50.0%) 2	2 (22.2%) 3	1 (20.0%) 1	1 (20.0%) 1	1 (33.3%) 3	3 (23.1%) 5
White blood cell count decreased	1 (20.0%) 1	1 (20.0%) 1	1 (33.3%) 1	3 (23.1%) 3	–	–	1 (50.0%) 2	1 (11.1%) 2	1 (20.0%) 1	1 (20.0%) 1	1 (33.3%) 3	3 (23.1%) 5
**Metabolism and nutrition disorders**	–	–	1 (33.3%) 1	1 (7.7%) 1	2 (66.7%) 2	3 (75.0%) 3	–	5 (55.6%) 5	2 (40.0%) 2	3 (60.0%) 3	1 (33.3%) 1	6 (46.2%) 6
**Skin and subcutaneous tissue disorders**	–	2 (40.0%) 2	1 (33.3%) 1	3 (23.1%) 3	–	2 (50.0%) 2	1 (50.0%) 1	3 (33.3%) 3	–	4 (80.0%) 4	1 (33.3%) 2	5 (38.5%) 6
Rash	–	2 (40.0%) 2	–	2 (15.4%) 2	–	–	1 (50.0%) 1	1 (11.1%) 1	–	2 (40.0%) 2	1 (33.3%) 1	3 (23.1%) 3

	Onset Before MDG1011 Administration (SAF)				Onset After Beginning of MDG1011 Administration (SAF-MDG1011)				Entire Study (SAF)			
	**Cohort 1**	**Cohort 2**	**Cohort 3**	**All Patients**	**Cohort 1**	**Cohort 2**	**Cohort 3**	**All Patients**	**Cohort 1**	**Cohort 2**	**Cohort 3**	**All Patients**
**System Organ Class**	**N = 5**	**N = 5**	**N = 3**	**N = 13**	**N = 3**	**N = 4**	**N = 2**	**N = 9**	**N = 5**	**N = 5**	**N = 3**	**N = 13**
**Gastrointestinal disorders**	2 (40.0%) 4	1 (20.0%) 3	1 (33.3%) 1	4 (30.8%) 8	1 (33.3%) 1	1 (25.0%) 1	–	2 (22.2%) 2	2 (40.0%) 5	1 (20.0%) 4	1 (33.3%) 1	4 (30.8%) 10
Nausea	2 (40.0%) 2	1 (20.0%) 1	–	3 (23.1%) 3	–	–	–	–	2 (40.0%) 2	1 (20.0%) 1	–	3 (23.1%) 3
Constipation	–	–	1 (33.3%) 1	1 (7.7%) 1	1 (33.3%) 1	–	–	1 (11.1%) 1	1 (20.0%) 1	–	1 (33.3%) 1	2 (15.4%) 2
Vomiting	1 (20.0%) 1	1 (20.0%) 1	–	2 (15.4%) 2	–	–	–	–	1 (20.0%) 1	1 (20.0%) 1	–	2 (15.4%) 2
**Neoplasms benign, malignant and unspecified (incl. cysts and polyps)**	1 (20.0%) 1	–	1 (33.3%) 1	2 (15.4%) 2	–	1 (25.0%) 1	1 (50.0%) 1	2 (22.2%) 2	1 (20.0%) 1	1 (20.0%) 1	2 (66.7%) 2	4 (30.8%) 4
Acute myeloid leukemia[a]	–	–	1 (33.3%) 1	1 (7.7%) 1	–	–	1 (50.0%) 1	1 (11.1%) 1	–	–	2 (66.7%) 2	2 (15.4%) 2
**Respiratory, thoracic and mediastinal disorders**	1 (20.0%) 1	1 (20.0%) 3	–	2 (15.4%) 4	–	2 (50.0%) 3	–	2 (22.2%) 3	1 (20.0%) 1	3 (60.0%) 6	–	4 (30.8%) 7
Dyspnoea	1 (20.0%) 1	1 (20.0%) 1	–	2 (15.4%) 2	–	1 (25.0%) 1	–	1 (11.1%) 1	1 (20.0%) 1	2 (40.0%) 2	–	3 (23.1%) 3
**Cardiac disorders**	1 (20.0%) 1	–	–	1 (7.7%) 1	1 (33.3%) 1	1 (25.0%) 1	–	2 (22.2%) 2	2 (40.0%) 2	1 (20.0%) 1	–	3 (23.1%) 3
**Injury, poisoning and procedural complications**	–	–	1 (33.3%) 1	1 (7.7%) 1	–	2 (50.0%) 2	–	2 (22.2%) 2	–	2 (40.0%) 2	1 (33.3%) 1	3 (23.1%) 3
**Musculoskeletal and connective tissue disorders**	–	2 (40.0%) 2	–	2 (15.4%) 2	1 (33.3%) 1	–	–	1 (11.1%) 1	1 (20.0%) 1	2 (40.0%) 2	–	3 (23.1%) 3
**Nervous system disorders**	–	2 (40.0%) 2	–	2 (15.4%) 2	–	3 (75.0%) 6	–	3 (33.3%) 6	–	3 (60.0%) 8	–	3 (23.1%) 8
Dizziness	–	–	–	–	–	2 (50.0%) 3	–	2 (22.2%) 3	–	2 (40.0%) 3	–	2 (15.4%) 3
Headache	–	1 (20.0%) 1	–	1 (7.7%) 1	–	2 (50.0%) 2	–	2 (22.2%) 2	–	2 (40.0%) 3	–	2 (15.4%) 3
**Eye disorders**	–	1 (20.0%) 1	1 (33.3%) 1	2 (15.4%) 2	–	1 (25.0%) 1	1 (50.0%) 1	2 (22.2%) 2	–	1 (20.0%) 2	1 (33.3%) 2	2 (15.4%) 4
**Immune system disorders**	–	–	–	–	–	1 (25.0%) 1	1 (50.0%) 1	2 (22.2%) 2	–	1 (20.0%) 1	1 (33.3%) 1	2 (15.4%) 2
Cytokine release syndrome	–	–	–	–	–	1 (25.0%) 1	1 (50.0%) 1	2 (22.2%) 2	–	1 (20.0%) 1	1 (33.3%) 1	2 (15.4%) 2

N = number of patients in the analysis set; n = number of patients in the category; nae = number of adverse events; TEAE = treatment-emergent adverse event; SOC = system organ class; PT = preferred term.

[a] Progression of the study disease was also to be reported as adverse even
